# Molecular characterization of a putative serine protease from *Trichinella spiralis* and its elicited immune protection

**DOI:** 10.1186/s13567-018-0555-5

**Published:** 2018-07-13

**Authors:** Ge Ge Sun, Hua Nan Ren, Ruo Dan Liu, Yan Yan Song, Xin Qi, Chen Xi Hu, Fan Yang, Peng Jiang, Xi Zhang, Zhong Quan Wang, Jing Cui

**Affiliations:** Department of Parasitology, Medical College, Zhengzhou University, Zhengzhou, 450052 China

## Abstract

In our previous work, a *Trichinella spiralis* putative serine protease (TsSP) was identified from ES products of *T. spiralis* intestinal infective larvae (IIL) and adult worms (AW) by immunoproteomics: it was highly expressed in IIL compared with muscle larvae (ML). In this study, the TsSP biological characteristics in larval invasion and growth were identified and its potential as a vaccine target against *Trichinella* infection were investigated. Expression of TsSP at various developmental phases (newborn larvae, ML, IIL, and AW) was detected by qPCR, immunofluorescent test and Western blotting. The rTsSP could specifically bind to the intestinal epithelial cell (IEC) membrane and enter into the cytoplasm. Anti-rTsSP serum suppressed the larval invasion of enterocytes in a dose-dependent mode, and killed newborn and ML of *T. spiralis*, decreased larval infectivity and development in the host by an ADCC-mediated mechanism. Immunization of mice with rTsSP produced a Th2 predominant immune response, and resulted in a 52.70% reduction of adult worms at 5 days post-infection (dpi) and a 52.10% reduction of muscle larvae at 42 dpi. The results revealed there was an interaction between TsSP and the host’s IEC; TsSP might be a pivotal protein for the invading, growing and parasiting of this nematode in the host. Vaccination of mice with rTsSP elicited immune protection, and TsSP is a potential target molecule for vaccines against enteral *Trichinella* infection.

## Introduction

Trichinellosis is an important zoonotic parasitic disease worldwide, especially in developing countries [1]. *Trichinella* infection results from the ingestion of raw or poorly cooked meat containing the larvae of the genus *Trichinella* [2]. *Trichinella* infection in animals and humans is recorded in 66 countries all over the world, and has been regarded as an emerging or re-emerging zoonotic disease [3]. *Trichinella spiralis* is the major etiological agent of *Trichinella* infection in humans, and its main reservoir is domestic pigs [4]. In China, 15 outbreaks of human trichinellosis were reported during 2004–2009, pork and pork-related products were the main sources of infection [5–7]. *Trichinella* infection in domestic pigs is a major public hygiene problem and hazard animal food safety [8]. There are increasing cosmopolitan demands for reliable preventive measures for *Trichinella* infection in food animals to ensure meat safety [9]. Therefore, the exploitation of vaccines to prevent domestic swine from *Trichinella* infection is a promising measure for the control of this zoonosis [10–13].

The lifecycle of *T. spiralis* is completed in a single host, the adult worms (AW) and larvae reside in the same host’s intestine and skeletal muscles, respectively. Once the contaminated meat is ingested, the muscle larvae (ML) of *T. spiralis* are liberated from the capsules, migrate to the small intestine, and develop into intestinal infective larvae (IIL) upon activation by bile [14, 15]. The IIL invade the small intestinal epithelium, undergo four molts and finally develop into adults. The AW copulate and adult females begin depositing the newborn larvae (NBL) at 5 days post-infection (dpi). Then, the NBL enter blood circulation, penetrate into skeletal muscles and encapsulate, and the lifecycle is accomplished [16]. However, the mechanism of invasion of enteral epithelium by *T. spiralis* IIL is still unknown. The larval surface or excretory–secretory (ES) proteases might participate in larval invasion of enteral epithelium [17–19]. Previous studies revealed that the IIL generates several proteases and invades the cell monolayer while they co-culture with the intestinal epithelial cell (IEC) monolayer [20, 21]. These proteases might interact with IEC and act as a principal part in the process of larval invasion of intestinal mucosa, and they are likely the potential vaccine targets against intestinal *Trichinella* infection.

In our previous studies, a putative serine protease of *T. spiralis* (TsSP) (GenBank accession no. ABY60762) was identified in ES products from *T. spiralis* IIL and AW by immunoproteomics with early infection sera from mice and patients with trichinellosis [19, 22]. Moreover, the TsSP gene was highly expressed in the IIL stage compared with the ML stage [18]. The complete TsSP cDNA sequences were cloned and expressed in our department [22, 31]. Sequence analysis showed that the complete cDNA sequences of TsSP gene were 1236 bp. The TsSP open reading frame (ORF) encodes a 45.2 kDa protein of 411 amino acids. The signal peptide is located between 1–18 aa. The TsSP has a domain of trypsin-like serine protease carrying an active site of classic catalytic triad (Serine–Histidine–Aspartat) for proteolysis.

In this study, the biological property and function of TsSP in *T. spiralis* invasion and development were characterized, and the immune protection generated by immunization with the rTsSP was also evaluated in a mouse model.

## Materials and methods

### Parasites and experimental animals

*Trichinella spiralis* isolate (ISS534) was acquired from a naturally infected pig in Henan Province of China. This isolate was passaged at an interval of each 6 months in BALB/c mice in our department. Female, 6–8 weeks old BALB/c mice were obtained from the Experimental Animal Center of Zhengzhou University.

### Collection of worms and protein preparation

The ML was obtained by artificially digesting mouse carcasses experimentally infected with *T. spiralis* at 42 dpi [23, 24]. The IIL were separated from the intestines of mice infected orally with 5000 ML at 6 hours post-infection (hpi), and AW was isolated from the intestines at 3 and 6 dpi, respectively. The NBL were recovered from the pregnant females at 6 dpi and cultured at 37 °C for 24 h [25]. These worms were respectively added into 20 mM pH 7.2 Tris–HCl buffers, supplemented with protease inhibitors and sonicated (99 cycles of 5 s, and lapse times of 5 s at 0 °C). The lysates were centrifuged at 4 °C and 10 000 *g* for 20 min. The soluble crude proteins and excretory/secretory (ES) proteins of ML, IIL and AW were prepared [26]. The concentration of worm protein was assayed as reported previously [27].

### Cell culture and protein preparation

The IEC was prepared from fetal mouse intestines and used for the in vitro larval invasion assay [28]. The C2C12 cell (mouse striated muscle myoblast) was resistant to invasion and utilized as the negative control [29]. The cells were cultivated in Dulbecco’s modified Eagle’s medium and collected by trypsinization. The IEC lysate proteins were prepared by grinding, sonicating and centrifuging [30].

### Immunization of mice with the rTsSP

The complete TsSP cDNA sequences were cloned, and the recombinant plasmid pQE-80L/TsSP was transformed into *Escherichia coli* BL21 (DE3) (Novagen, USA). The rTsSP protein expression was induced for 4 h at 30 °C with 0.5 mM IPTG in our laboratory [31]. The rTsSP was purified using a Ni–NTA His-tag affinity kit (Novagen). A total of 60 BALB/c mice were randomly divided into three groups (20 mice per group). The experimental group of mice was immunized with rTsSP. Each mouse was immunized with abdominal subcutaneous injection with 20 μg of rTsSP emulsified in Freund’s complete adjuvant, subsequently boosted twice at a 10 days interval using the same dose of rTsSP emulsified with Freund’s incomplete adjuvant [32]. The control group was vaccinated with only adjuvant or PBS. Ten days post the ultimate boost, all mice were orally inoculated with 300 *T. spiralis* ML. Blood samples from tail veins of vaccinated mice were collected at 0, 10, 20, 30 and 40 days after immunization [33]. Pre-immune serum was also collected and utilized as negative serum control.

### Real-time quantitative PCR (qPCR)

Total RNA from diverse *T. spiralis* phases (NBL, ML, IIL and AW) were prepared using TRIzol reagent (Invitrogen). In order to eliminate the contamination with DNA, all RNA were pre-treated with DNase I (Thermo Fisher Scientific, San Francisco, CA, USA). qPCR was carried out as described [34]. The 20 μL of reaction mixture consisted of 1× iTaq Universal SYBR^®^ Green (Bio-Rad Laboratories, Inc.), 2 μL of the first-strand cDNA, and 300 nM each of the Fwd and Rev Primer. The primer for TsSP qPCR was as follows: 5′-CTTTTCAAGTGCTTATTTCTC-3′ and 5′-TATTACCCGCTTTTCTGAAC-3′. The TsSP transcription level was standardized by subtracting the transcription level of Glyceraldehyde 3-phosphate dehydrogenase (G3PDH) of the same group. qPCR amplification was conducted using LightCycler^®^ 480 II Real-Time PCR System (Roche Applied Science, Mannheim, Germany), according to the following procedures: 95 °C for 30 s; 40 cycles of 95 °C for 15 s and finally 60 °C for 1 min. A melting curve analysis was carried out at 65–95 °C. The TsSP transcription levels were calculated on the basis of a 2^−ΔΔCt^ formula [18]. The experiment was performed using three replicates and each sample had three replicates.

### TsSP expression in various stages identified by Western blot analysis

Soluble and ES proteins from various stages of *T. spiralis* (NBL, ML, IIL and AW) were identified by Western blot analysis with anti-rTsSP serum. Protein samples were separated by SDS-PAGE in 12% polyacrylamide gels. After electrophoresis, the gels were transferred to nitrocellulose membranes (Merck Millipore, Billerica, MA, USA) [35]. Membranes were blocked at 37 °C for 2 h with 5% skimmed milk in Tris–buffered saline with Tween-20 (TBST). The membranes were washed three times and incubated with 1:100 dilutions of anti-rTsSP serum. After being washed again, the membranes were further incubated with HRP-conjugated goat anti-mouse IgG (1:10 000). The coloration was developed using 3,3′-diaminobenzidine tetrahydrochloride (DAB; Sigma, USA) and terminated by washing the membrane with deionized water.

### Immunostaining of TsSP at various stages by immunofluorescent test (IFT)

Immunostaining of TsSP at various *T. spiralis* whole worm stages (NBL, ML, IIL, AW and embryos) and 3-μm cryosections of ML and AW was performed as described [36, 37]. Briefly, the intact worm was immersed in 100% cold acetone for 20 min. The worms were spread on polylysine-coated slides, permeabilized using 1% Triton X-100 for 5 min, and blocked with 3% bovine serum albumin (BSA). Adult females were incised from their middle to release embryos on slides, and the embryos were covered with a cover slip. The slides were first put into liquid nitrogen, and then fixed with 100% cold acetone for 20 min after the cover slip was taken out. After washing, the whole worms and sections were blocked at 37 °C for 2 h with 3% BSA, reacted with 1:10 dilutions of anti-rTsSP serum, and stained at 37 °C for 1 h with 1:100 dilutions of cy3/FITC-conjugated goat anti-mouse-IgG. Subsequently, the whole worms were incubated with a 4′,6-diamidino-2-phenylindole (DAPI) at 37 °C for 5 min. Finally, the slides were treated with 70% glycerol and observed with a fluorescent microscope (Olympus, Japan).

### Determination of rTsSP-specific antibodies by ELISA

Serum anti-rTsSP antibodies (total IgG, IgG1 and IgG2a) of vaccinated mice were assayed by ELISA at 10 days following each vaccination [38, 39]. Briefly, the plates were coated with 2 μg rTsSP/mL and incubated with mouse sera diluted at 1:100 in PBST. After being washed, the plate was incubated with 1:10 000 dilutions of HRP-conjugated goat anti-mouse IgG (or IgG1, IgG2a (Sigma-Aldrich), then with o-phenylenediamine dihydrochloride (OPD; Sigma-Aldrich). A microplate reader (Tecan, Schweiz, AG, Switzerland) was applied to measure the absorbance at 490 nm.

### Assay of binding activity of rTsSP with IEC by far Western

For far Western analysis of rTsSP and IEC interaction, the protein samples of IEC and C2C12 were separated with 12% polyacrylamide gels for 2.5 h [40]. The gels were transferred to nitrocellulose membranes (Millipore, USA). Then the blots were blocked for 2 h at 37 °C by 5% nonfat milk in TBST. The blots were incubated (37 °C, 2 h) with 20 μg/mL rTsSP, and the IIL ES proteins as the control group [30]. Membranes were washed three times and incubated (37 °C, 2 h) with 1:100 dilutions of different sera (anti-rTsSP serum, pre-immune serum or infection serum). After being washed completely, the membranes were incubated at 37 °C for 1 h with HRP-conjugated anti-mouse IgG, and stained using DAB (Sigma-Aldrich). The IEC protein bands bound with rTsSP were analyzed with the AlphaView Software.

### Determination of binding between rTsSP and IEC by ELISA

The binding ability between rTsSP and IEC was also investigated by ELISA [11, 41]. Briefly, the plate was coated with different concentrations of IEC proteins. After being blocked and washed, the following reagents were successively added: different dilutions of the rTsSP, 1:100 dilutions of mouse sera, and HRP-conjugated goat anti-mouse IgG, (1:10 000; Sigma-Aldrich). The plate was respectively incubated at 37 °C for 2 h. After being reacted with OPD, the absorbance at 490 nm was measured as before. All samples were run in duplicate.

### Binding and cellular localization of rTsSP with IEC by IFT

The IEC and C2C12 cell was grown to confluence on glass coverslips in DMEM medium. The monolayer was incubated with 20 μg/mL rTsSP at 37 °C 2 h. The IIL ES protein and PBS were respectively utilized as a positive or negative control [30]. After being washed, the monolayer was fixed with 4% formaldehyde for 20 min. The monolayer was incubated with 1:10 dilutions of anti-rTsSP serum, infection serum or pre-immune serum, then incubated with FITC-conjugated goat anti-mouse IgG diluted at 1:100 (Santa Cruz, USA) for 45 min at 37 °C. Finally, the monolayer was incubated for 5 min with propidium iodide (PI) to specifically stain the cell nucleus. Upon completion of the staining procedure, the coverslips were transferred to glass slides and observed with fluorescent microscopy (Olympus, Tokyo, Japan) [33]. Moreover, cellular localization of rTsSP within enterocytes was further examined with confocal microscopy and analyzed with Olympus Fluoview software [30].

### The in vitro invasion assay

The IIL was applied for the in vitro invasion assay [15]. The IEC monolayers were overlaid with 200 IIL suspended in semisolid medium with different dilutions of anti-rTsSP serum, infection serum or pre-immune serum, and incubated in 5% CO_2_ for 2 h at 37 °C [29, 42]. The number of larvae invaded within IEC monolayers was counted with an inverted phase-contrast microscope (Olympus, Japan). The IIL penetrated into and migrated in the monolayer were calculated as invaded IIL; when the IIL were still suspended in medium, they were assessed as non-invading IIL [26]. Each invasion assay had three replicates.

### In vitro antibody-dependent cellular cytotoxicity (ADCC) assay

Anti-rTsSP antibody cytotoxicity on *T. spiralis* NBL and ML was tested as described [43, 44]. Pre-immune and infection serum were respectively utilized as negative or positive controls. Peritoneal macrophages (PM) were obtained from normal mice. One hundred of NBL (or 50 ML) were added into RPMI 1640 media containing 2 × 10^5^ PM and 1:5–1:1000 dilutions of anti-rTsSP serum. Then, the plate was incubated in 5% CO_2_ at 37 °C for 72 h. Each measurement was done in triplicate. Worm viability after the ADCC assay was assessed according to their morphology and activity under a microscope. The live NBL and ML were mobile and exhibited wriggling motion; whereas the dead NBL were straight, inactive or disintegrated, the dead ML were immobile and straight as “C” shapes [42]. Additionally, to identify the viability and infectivity of the ML in ADCC assay, each mouse from three groups of mice was inoculated orally with 100 ML treated by ADCC assay for 24 h with 1:100 dilutions of different serum. The cytotoxicity of anti-rTsSP antibodies on the ML was further evaluated on the basis of recovered intestinal AW at 3 dpi and larvae per gram (LPG) muscle collected from inoculated mice at 42 dpi [13, 35].

### Challenge infection and protective efficacy evaluation

To evaluate the immune protection, three groups of mice were challenged orally with 300 ML of *T. spiralis* 10 days after the last immunization. Adults in the intestine were recovered from 10 mice at 5 dpi, and the ML in skeletal muscles was collected from another ten mice at 42 dpi. The worm reduction rates in AW and ML were evaluated in accordance with the number of collected intestinal AW and larvae per gram (LPG) muscles from the immunized mice with rTsSP relative to those from PBS control [11, 45].

### Statistical analysis

The statistical analysis of data was performed using SPSS 17.0 software. All data are shown as arithmetic mean ± standard deviation. One-way ANOVA or Student’s t-test was applied for comparison among different groups. The level of statistical significance was *P* < 0.05.

## Results

### qPCR analysis of TsSP gene transcription at different stages

**Figure 1:**
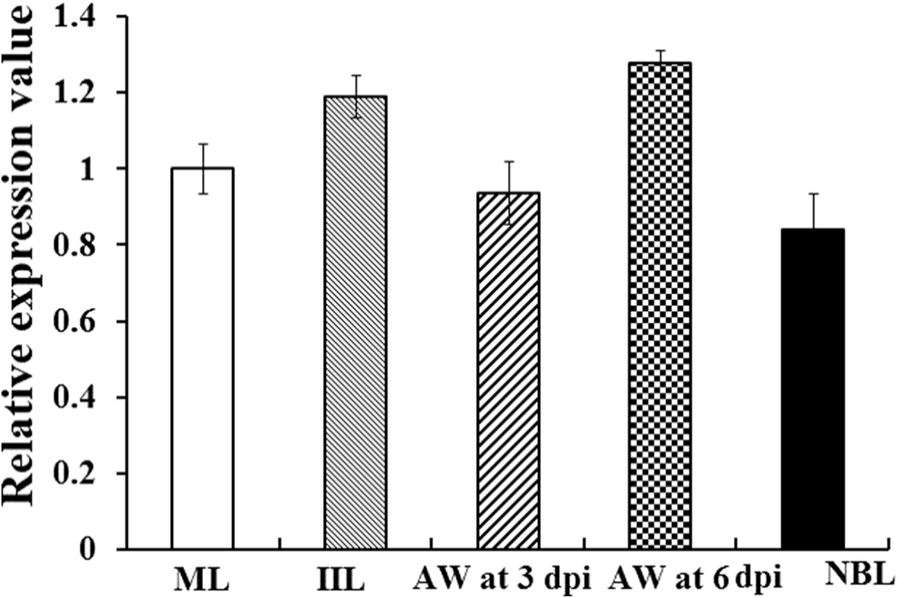
**qPCR analysis of TsSP transcription level at different stages** ***of T. spiralis*.** The TsSP mRNA from AW at 3 and 6 dpi, and NBL, ML and IIL were amplified by qPCR. The TsSP mRNA transcription levels were calculated via the Ct (2^−ΔΔCt^) method. G3PDH was utilized as a housekeeping gene control. TsSP transcription level was not statistically different among different stages of *T. spiralis* (*P* = 0.05).

The TsSP gene transcription in various stages of *T. spiralis* was determined by qPCR. In the life cycle of *T. spiralis*, the TsSP gene was transcribed at all developmental stages (NBL, ML, IIL, and AW) (Figure 1). The transcription level of the TsSP gene was not statistically different among different stages (*F *= 3.620, *P* = 0.05).

### Western blot analysis of TsSP expression in various stages

**Figure 2:**
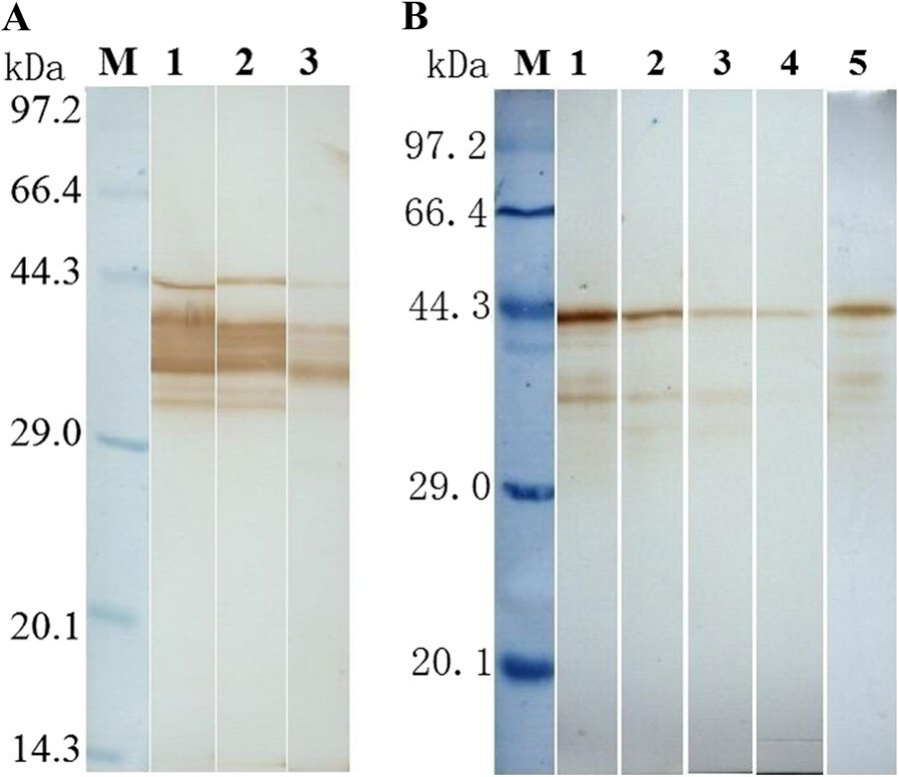
**Western blot analysis of TsSP expression in diverse** ***T. spiralis*** **phases. A** Western blotting of ES proteins of diverse *T. spiralis* phases using anti-rTsSP serum. Lane 1: ML; lane 2: IIL; lane 3: AW at 3 dpi. **B** Western blotting of soluble proteins of diverse *T. spiralis* phases using anti-rTsSP serum. Lane 1: ML; lane 2: IIL; lane 3: AW at 3 dpi; lane 4: AW at 6 dpi; lane 5: NBL.

The native TsSP protein with 25–47 kDa in ES proteins from *T. spiralis* IIL, AW at 3 dpi and ML was identified by anti-rTsSP serum using Western blotting (Figure 2A). Furthermore, the native TsSP protein in crude proteins from diverse *T. spiralis* phases (ML, IIL, AW and NBL) was also recognized by anti-TsSP serum (Figure 2B), indicating that the TsSP was expressed in all *T. spiralis* phases, and was an excretory or secretory protein.

### Immunolocalization of TsSP at various stages by IFT

**Figure 3:**
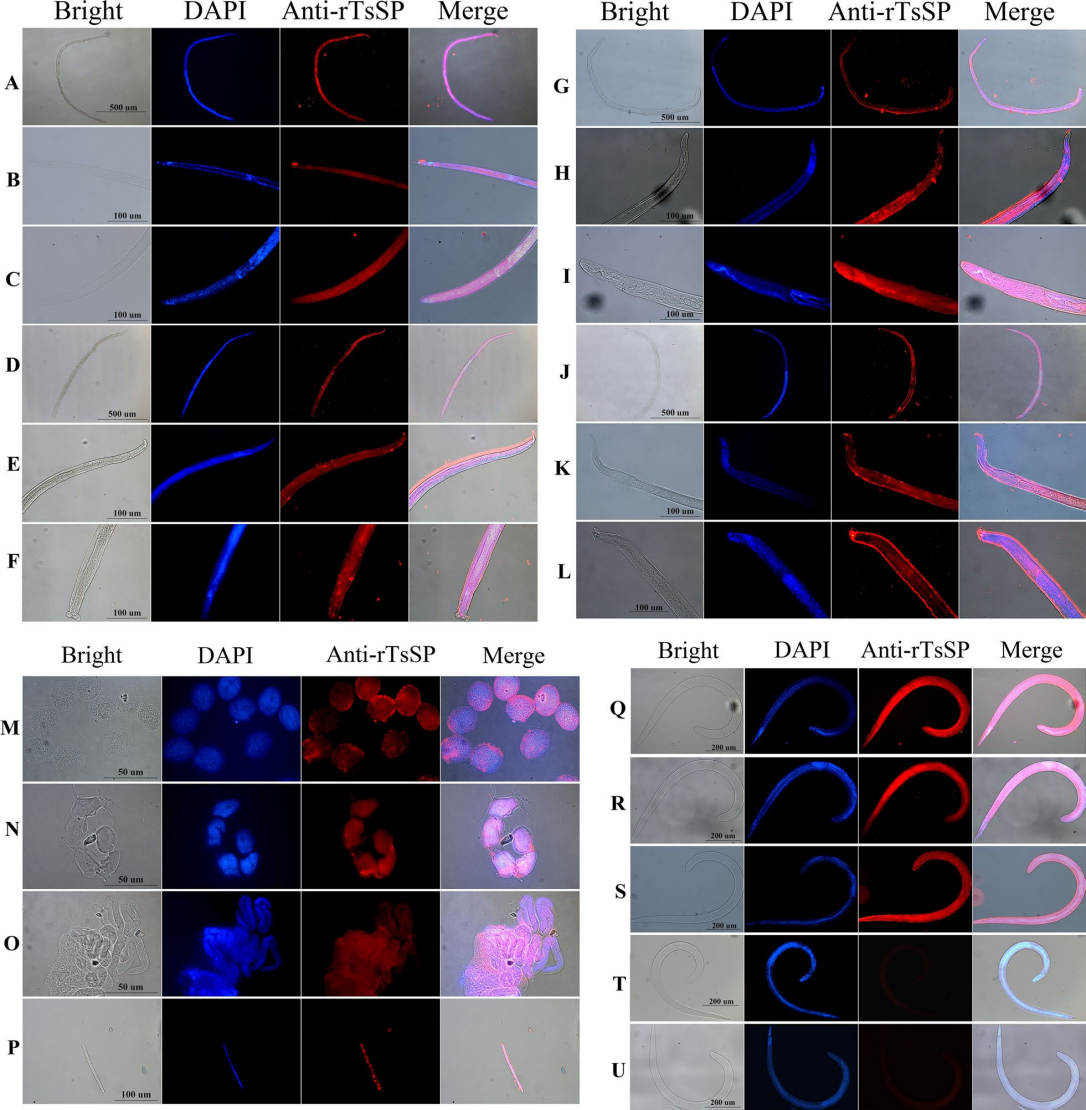
**Immunolocalization of TsSP in whole worms of** ***T. spiralis*** **various stages by IFT. A** Female AW at 3 dpi; **B** forepart of adult female at 3 dpi; **C** tail of adult female at 3 dpi; **D** adult male at 3 dpi; **E** forepart of adult male at 3 dpi; **F** tail of adult male at 3 dpi; **G** adult female at 6 dpi; **H** forepart of adult female at 6 dpi; **I** tail of adult female at 6 dpi; **J** adult male at 6 dpi; **K** forepart of adult male at 6 dpi; **L** tail of adult male at 6 dpi; **M** early embryos; **N** midterm embryos; **O** late embryos; **P** NBL; **Q** ML at 42 days; **R** IIL at 6 hpi; **S** ML recognized with infection serum was utilized as a positive control; ML incubated with pre-immune mouse serum (**T**) and PBS (**U**) as negative controls.

**Figure 4:**
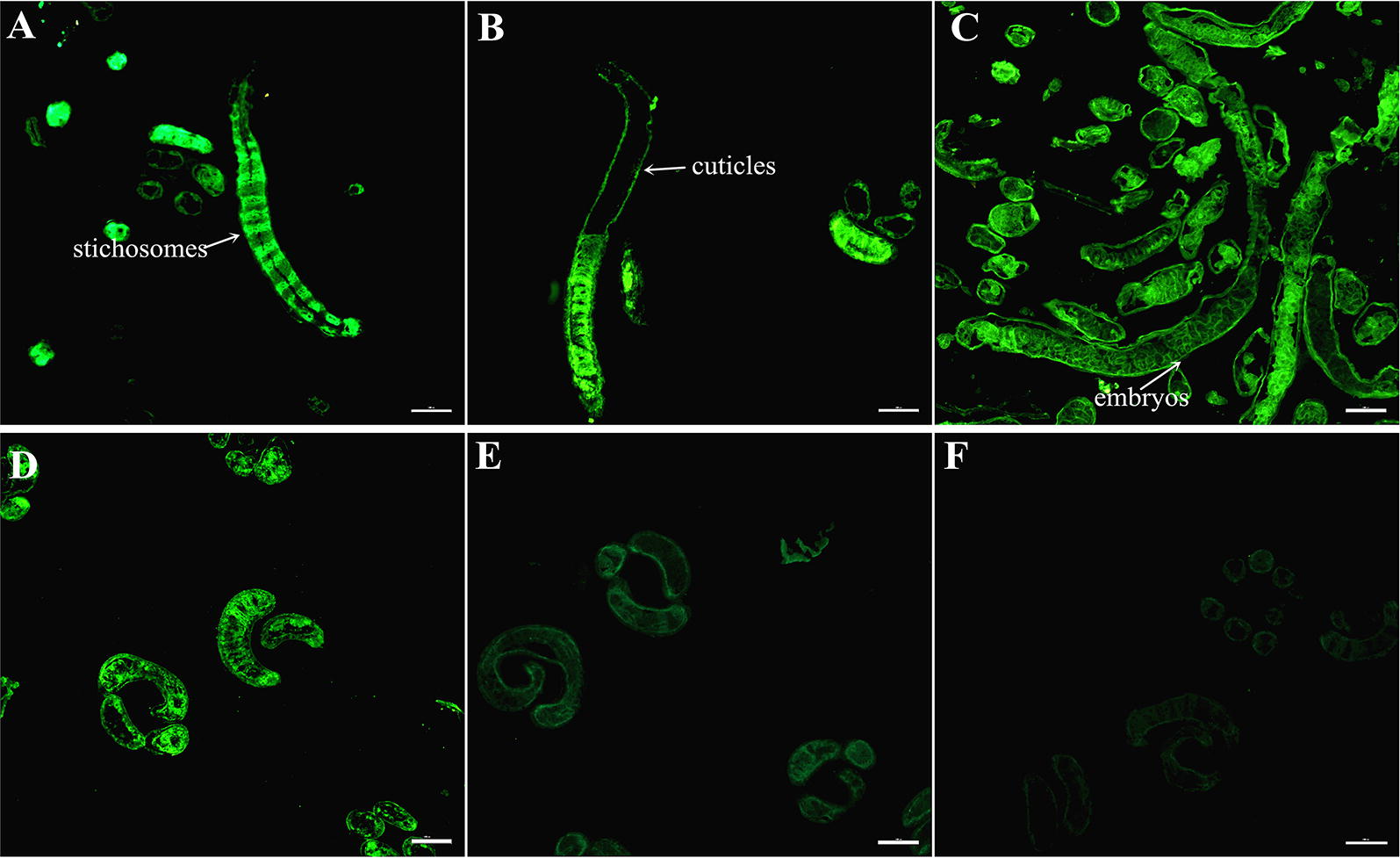
**Immunolocalization of TsSP by IFT with cryosections of** ***T. spiralis*** **ML and AW. A**, **B** Cryosections of the ML were probed with anti-rTsSP serum, and immunostaining was observed at the cuticle and stichosome of ML. **C** Cryosections of the 6 days old female adults were recognized by anti-rTsSP serum and the immunostaining was located in embryos of the females. **D** The ML recognized by mouse infection serum as a positive control. The ML not recognized by pre-immune serum (**E**) and PBS (**F**) as negative controls. Scale-bars: 100 μm.

Expression and immunolocalization of TsSP at different *T. spiralis* stages were investigated by IFT using anti-rTsSP serum, and immunostaining was found in cuticles and whole worm bodies of AW, all embryos (early, medium and late stages), NBL, ML and IIL. The staining of ML and IIL was brighter than those of AW and NBL (Figure 3). When the cryosections of the nematode were incubated with anti-rTsSP serum, the immunostaining was found at the cuticle, stichosome of ML, and embryos of 6 days old female worms (Figure 4). There was no immunostaining in the worms incubated by pre-immune serum or PBS.

### Binding of rTsSP to IEC analyzed by far Western analysis

**Figure 5:**
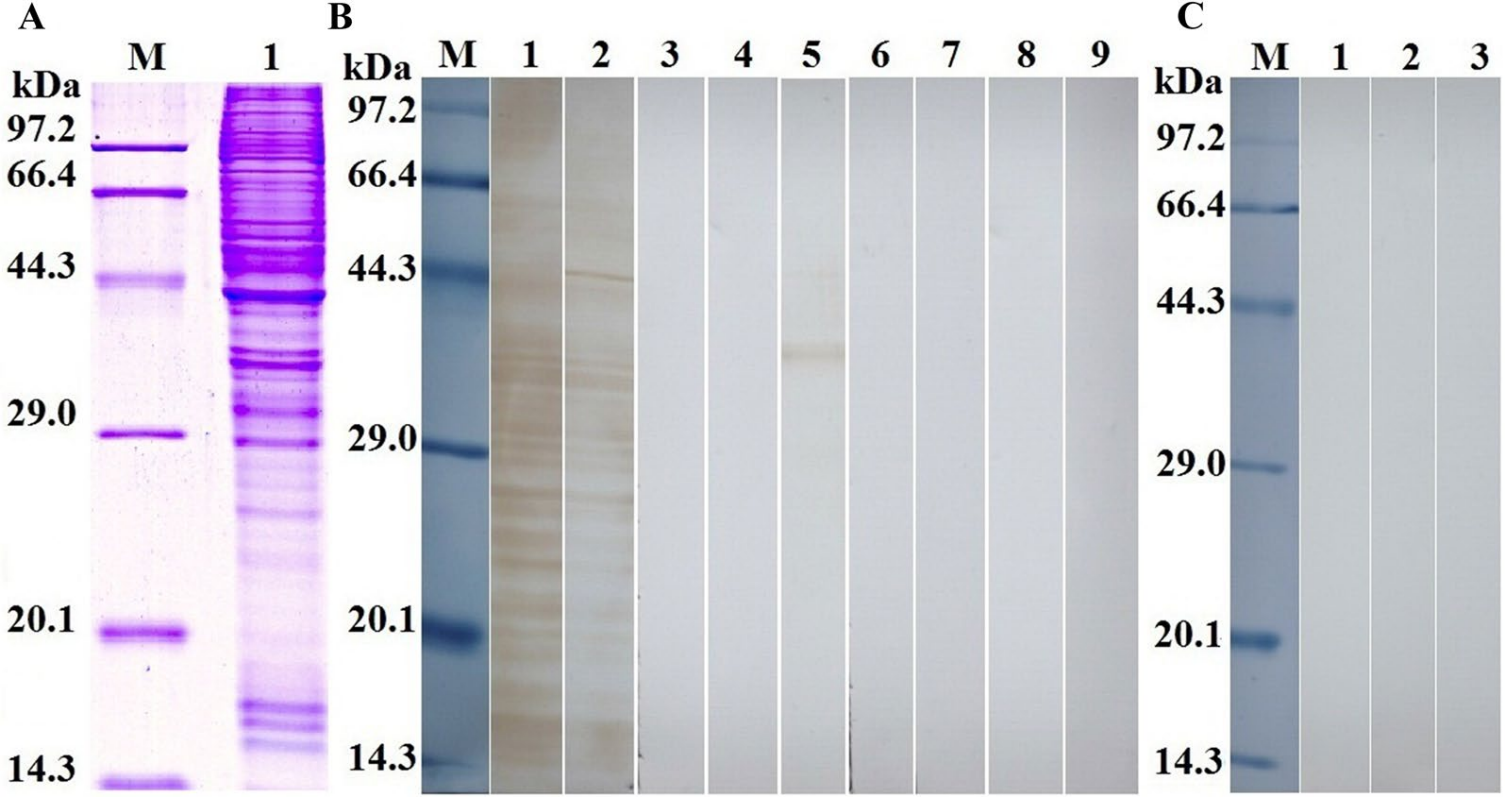
**Far Western analysis of rTsSP binding to IEC protein. A** SDS-PAGE of the IEC proteins. Lane M: the protein molecular weight marker; lane 1: the IEC lysate proteins. **B** Far-Western analysis of IEC proteins binding to rTsSP. The IEC protein was first reacted with rTsSP (lanes 1–3), IIL ES proteins (lanes 4–6) or PBS (lanes 7–9), and then recognized using anti-rTsSP serum (lanes 1, 4 and 7), infection serum (lanes 2, 5 and 8) or pre-immune serum (lanes 3, 6 and 9). **C** Far Western analysis of C2C12 protein binding to rTsSP. The C2C12 protein (lanes 1–3) was reacted first with rTsSP, and subsequently incubated with anti-rTsSP serum (lane 1), infection serum (lane 2) or pre-immune serum (lane 3). There was no binding between rTsSP and C2C12 protein.

The results of SDS-PAGE analysis showed that the IEC proteins had about 40 protein bands with a molecular weight of 14.4–97.2 kDa. Far Western results revealed that anti-rTsSP serum identified about 24 bands (14.4–86.5 kDa) of IEC lysates incubated with rTsSP, and infection serum recognized about 19 bands (14.4–58.7 kDa) (Figure 5A). The binding of IEC proteins with IIL ES protein was also identified by mouse infection sera, but not probed with anti-rTsSP serum, suggesting that the quantity of the native TsSP in IIL ES protein was too low to be detected by far Western. Pre-immune serum did not recognize the IEC proteins incubated with rTsSP (Figure 5B). No binding between C2C12 proteins and rTsSP was detected (Figure 5C). It demonstrated that the rTsSP had a specific binding and interaction with IEC.

### Determination of binding between IEC and rTsSP by ELISA

**Figure 6:**
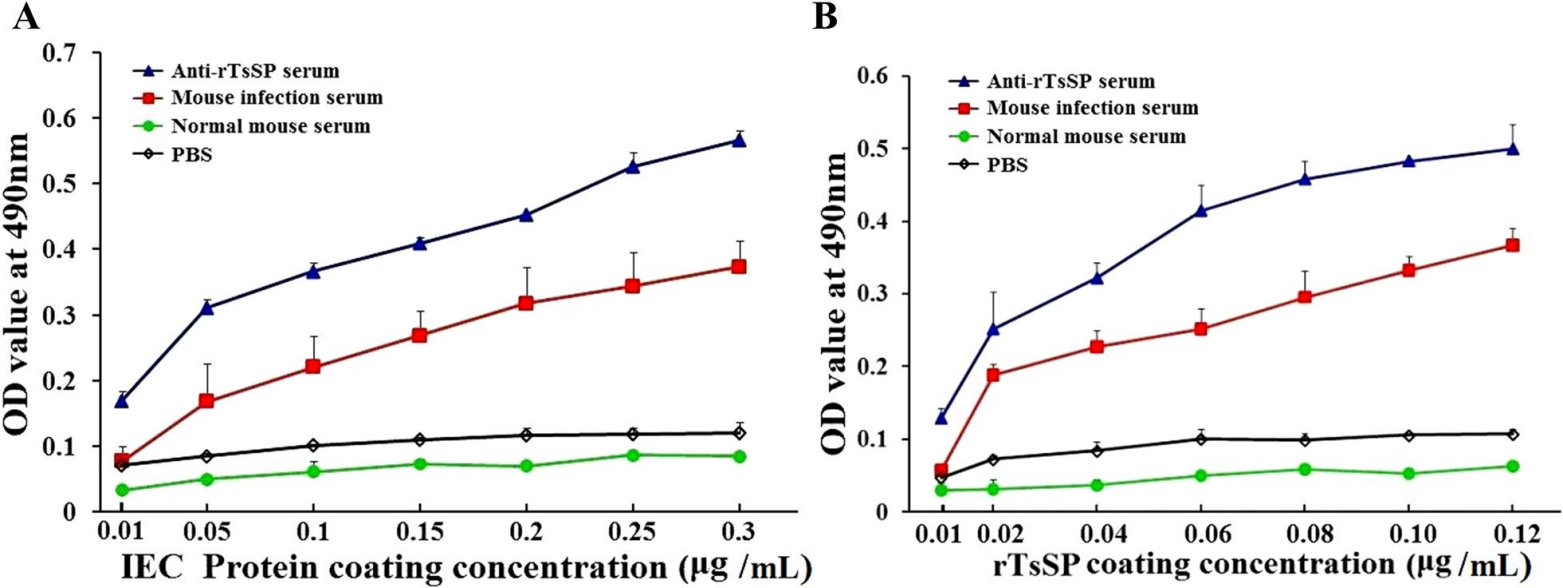
**Binding affinity between rTsSP and IEC assayed by ELISA. A** The binding between 5 μg/mL rTsSP and different concentrations of IEC proteins; **B** the binding between 2 μg/mL IEC proteins and different concentrations of rTsSP. The binding between rTsSP and IEC proteins is dose-dependent of IEC and rTsSP protein.

The binding affinity between rTsSP and IEC lysates was also assayed by ELISA. There was a strong in vitro interaction between rTsSP and IEC. The binding was IEC protein dose-dependent (*r* = 0.943, *P *< 0.01) and showed an increasing trend with an increase of IEC protein concentration (*F* = 1044.398, *P *< 0.01) (Figure 6A). Moreover, the binding was also rTsSP dose-dependent (*r *= 0.925, *P *< 0.01) and showed an increasing trend with an increase of rTsSP concentration (*F *= 360.641, *P *< 0.01) (Figure 6B).

### Binding and cellular localization of rTsSP with IEC by IFT

**Figure 7:**
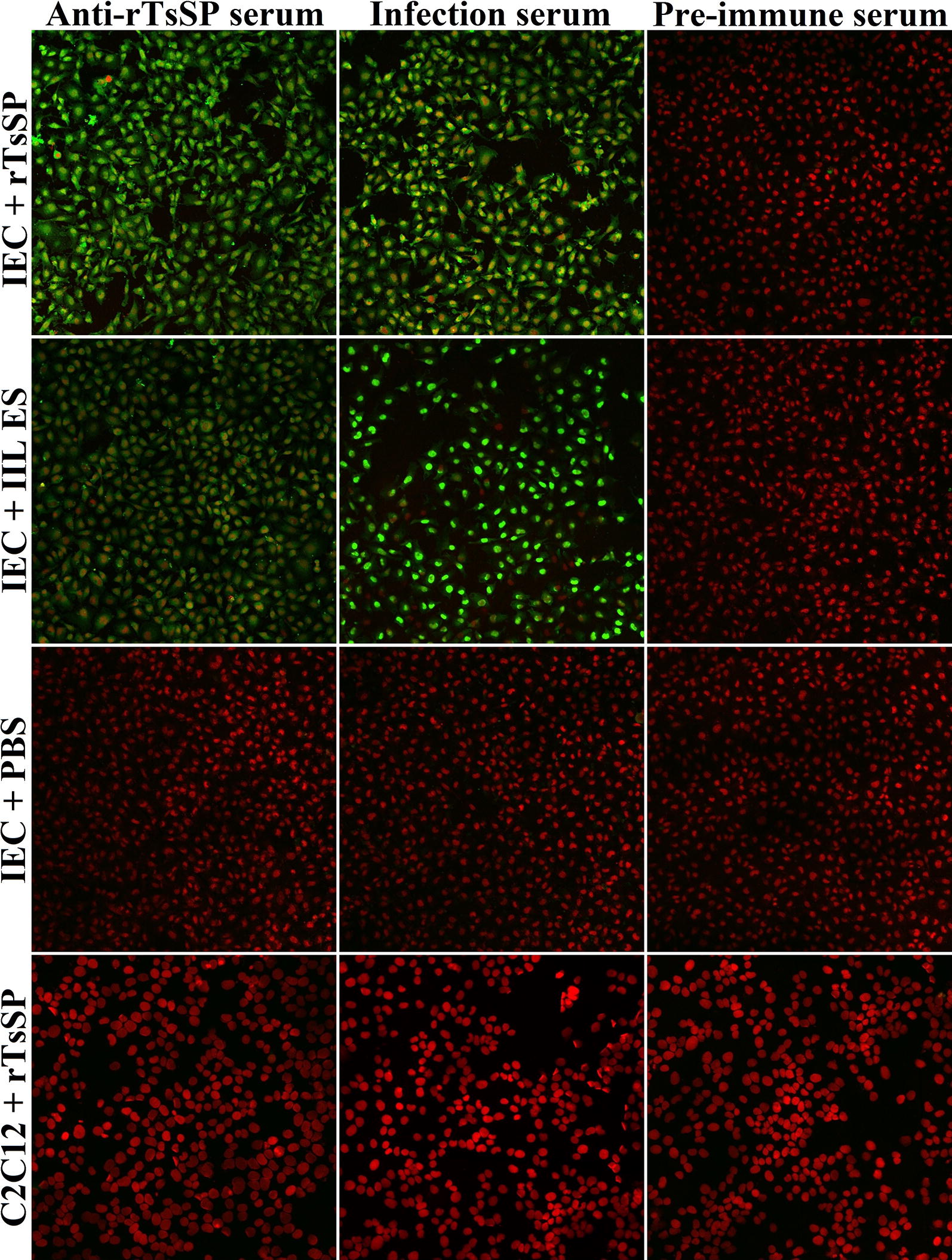
**Binding and cellular localization of rTsSP with IEC by IFT (×200).** The IEC was incubated with rTsSP, IIL ES proteins or PBS at 37 °C for 2 h. The C2C12 cell was also incubated with rTsSP at 37 °C for 2 h. Following washing, the IEC and C2C12 was incubated with anti-rTsSP serum, infection serum or pre-immune serum, and then colored with FITC-labeled goat anti-mouse IgG. Cell nuclei were stained with propidium iodide (PI) as red color.

**Figure 8:**
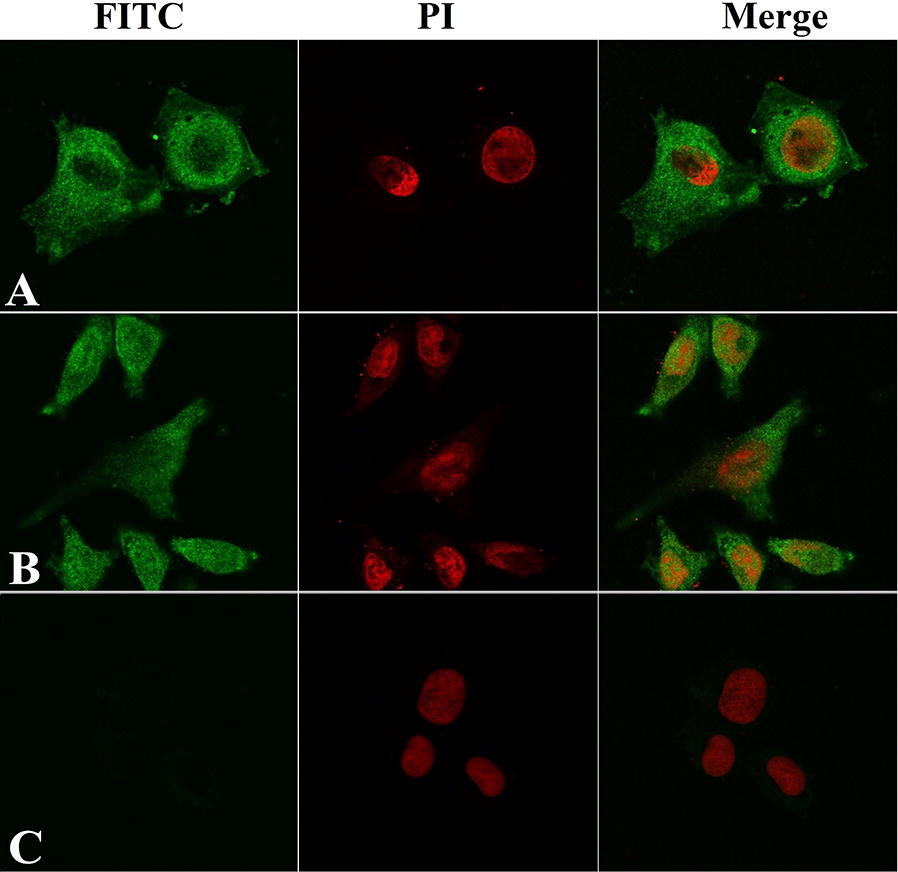
**Subcellular localization of rTsSP bonding to IEC by confocal microscopy (×1000).** The IEC was first incubated with rTsSP, secondly with anti-rTsSP serum (**A**), infection serum (**B**) or pre-immune serum (**C**), and finally with FITC-labeled goat anti-mouse IgG. Cell nuclei were stained using propidium iodide (PI) as red.

IFT results revealed that the immunostaining with anti-rTsSP serum or infection serum was observed on surfaces of IEC incubated with rTsSP. The positive staining using anti-rTsSP serum or infection serum was also seen on IEC incubated with IIL ES proteins. But, no staining of IEC incubated with rTsSP or IIL ES was detected using pre-immune serum. Furthermore, both anti-rTsSP serum and infection serum did not recognize the IEC incubated with PBS or C2C12 incubated with rTsSP (Figure 7). By confocal microscopy, immunostaining is mainly located in the IEC membrane and cytoplasm (Figure 8), demonstrating that rTsSP can bind to the IEC membrane and enter the cytoplasm.

### Inhibition of larval invasion of IEC by anti-rTsSP sera

**Figure 9:**
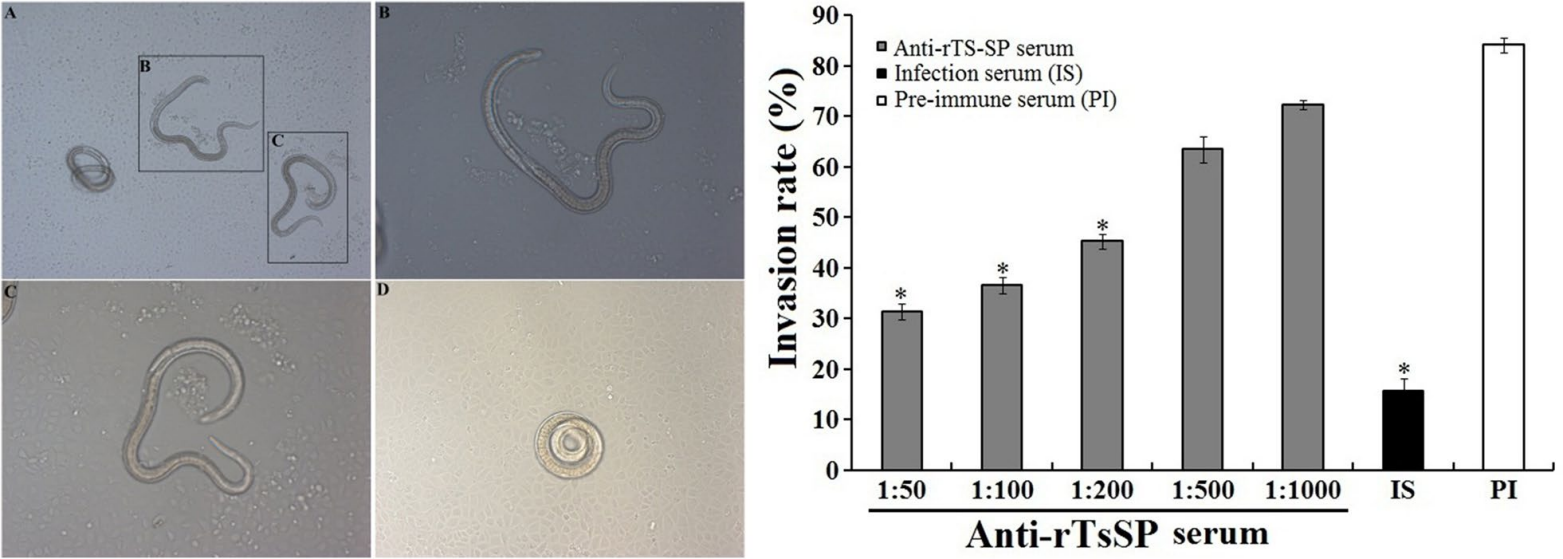
**The in vitro inhibition of** ***T. spiralis*** **invasion of IEC by anti-rTsSP serum.** Left: upon IIL addition to an IEC monolayer, the invaded and non-invaded larvae in monolayer (**A**, ×100). **B**, **C** Invaded larva in monolayer (×200). **D** (×200) Non-invaded and suspended larva in culture media (×200). Right: inhibition of larval invasion of IEC through various dilutions of anti-rTsSP serum. Infection serum (IS, 1:50 dilution) and pre-immune serum (PI, 1:50) were respectively utilized as a positive and negative control. The results are expressed as the percentages of the larvae invading the monolayer and they are shown as the mean ± SD of three independent experiments. Asterisks indicate remarkable differences (*P* < 0.01) relative to pre-immune serum.

When the IIL were added to an IEC monolayer, the larvae invaded the monolayer and migrated within it. When the culture medium was supplemented with 1:50 dilutions of anti-rTsSP serum, infection serum and pre-immune serum, the percentages of larvae invading the monolayer were 31.48, 15.84 and 84.16%, respectively (χ^2^ = 227.786, *P* < 0.01). The inhibition of anti-rTsSP serum on the IIL invasion of the IEC monolayer was significantly higher than that of the pre-immune serum (*χ*^2^ = 119.551, *P* < 0.01). The inhibition was dose-dependent and exhibited a decreasing trend along with a serum dilution increase (*F* = 198.787, *P* < 0.01). However, pre-immune serum had no obvious inhibition on larval invasion (Figure 9).

### In vitro ADCC assay

**Figure 10:**
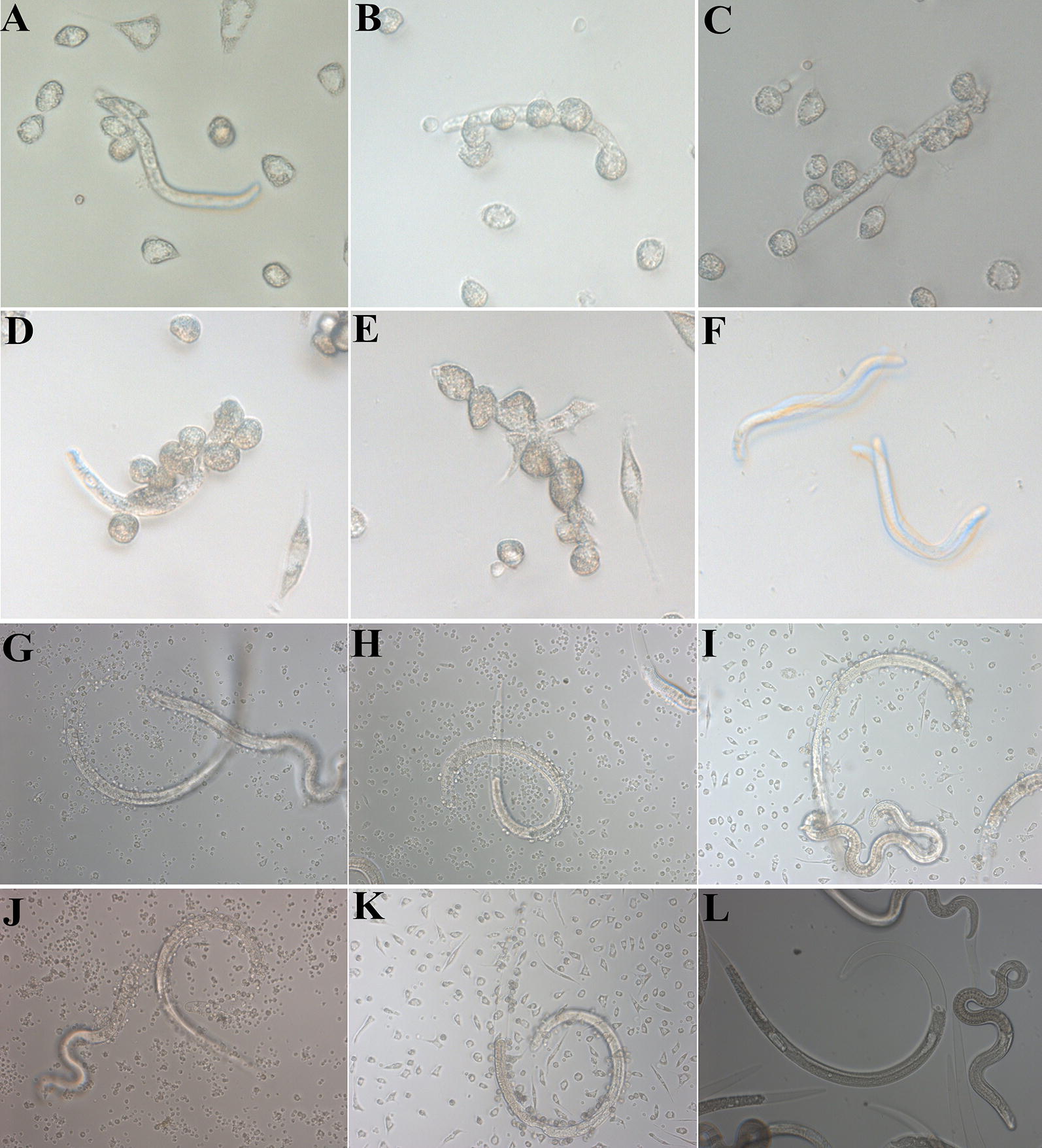
***T. spiralis*** **NBL and ML at different times after ADCC assay.** In the assay, the NBL (**A**–**F**) and ML (**G**–**L**) were cultured with anti-rTsSP serum and 2 × 10^5^ mouse peritoneal macrophages (PM) at 37 °C for different times, **A**, **G** 6 h; **B**, **H** 12 h; **C**, **I** 24 h; **D**, **J**: 48 h; **E**, **K**: 72 h; **F** (NBL) and **I** (ML): incubation without MPM for 72 h as a non-MPM control.

**Figure 11:**
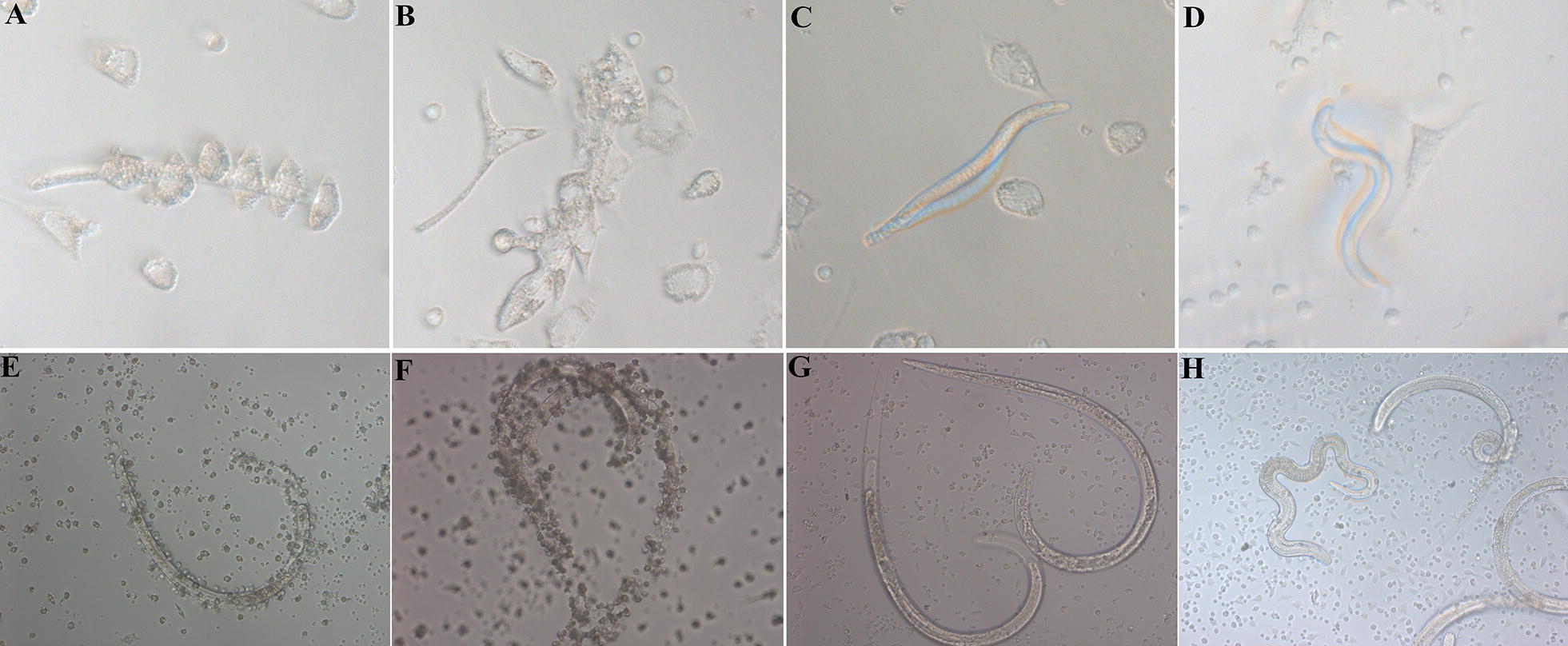
***T. spiralis*** **NBL and ML at 72** **h after ADCC with different serum.** In the assay, the NBL (**A**–**D**) and ML (**E**–**H**) were cultured at 37 °C for 72 h with 2 × 10^5^ mouse peritoneal macrophages (PM) and different mouse serum, **A**, **E** anti-rTsSP serum; **B**, **F** infection serum; **C**, **G** pre-immune serum; **D**, **H** PBS. No PM adhered to the live larvae cultured with pre-immune serum and PBS.

**Figure 12:**
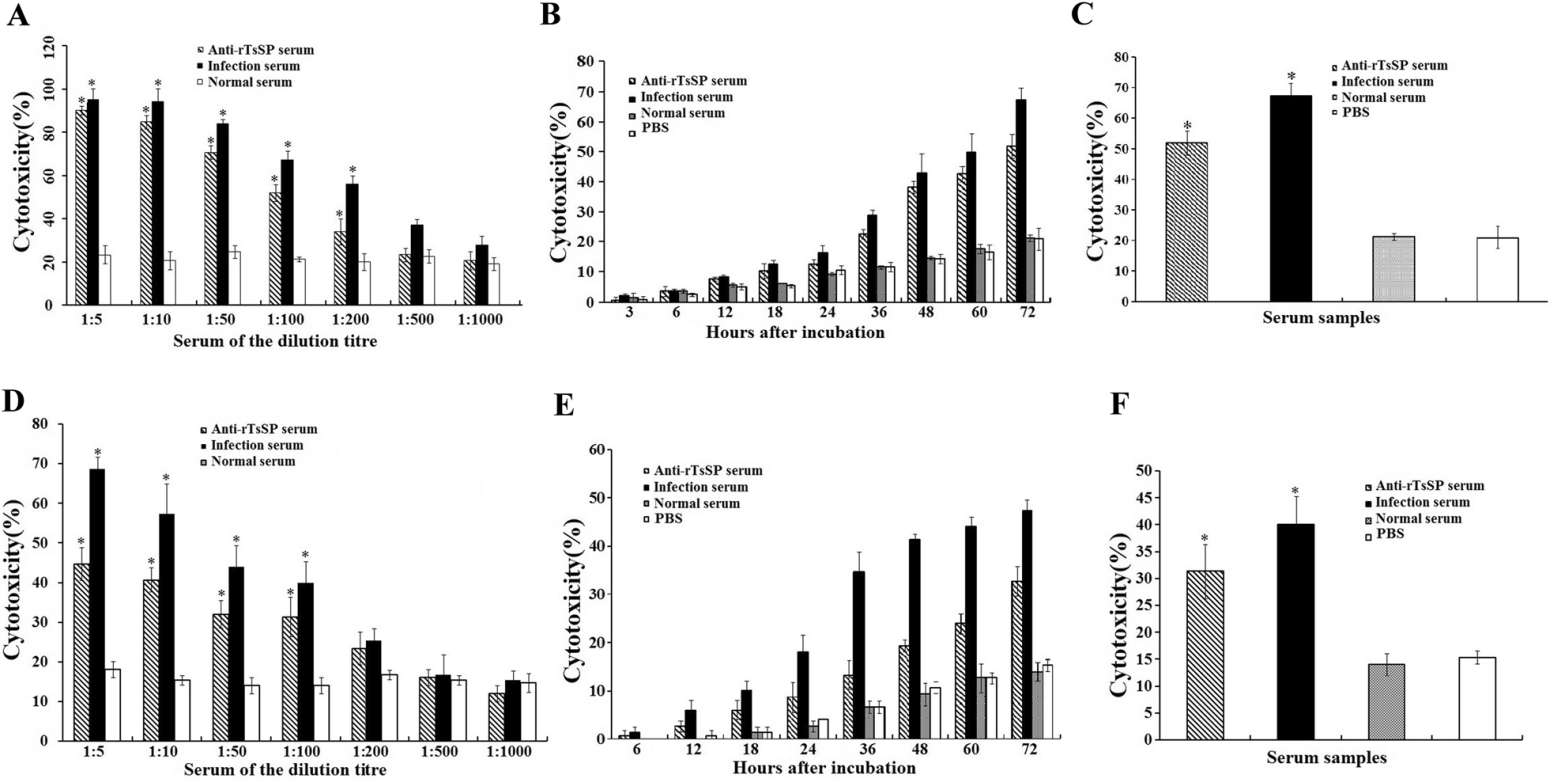
**ADCC assays of** ***T. spiralis*** **NBL (A**–**C) and ML (D**–**F). A**, **D** The cytotoxicity was anti-rTsSP antibody dose-dependent. **B**, **E** The cytotoxicity had an increasing trend with prolonged culture time. **C**, **F** ADCC assay with different serum. When 1:100 dilutions of different serum was used and cultured for 72 h, the ADCC with anti-rTsSP serum caused the evident death of NBL (**C**) and ML (**F**), compared with the larvae incubated by normal mouse serum (pre-immune serum). Asterisks show a statistically prominent difference (*P* < 0.01) in comparison with normal serum.

**Figure 13:**
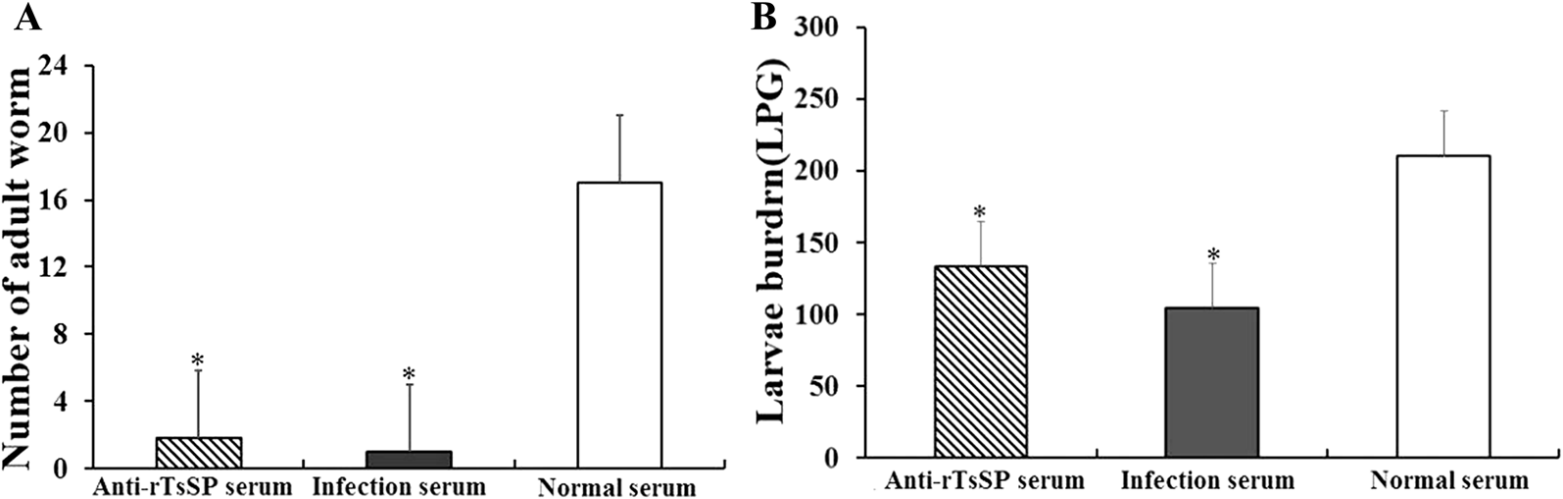
**Identification of the infectivity of** ***T. spiralis*** **ML treated by ADCC assay.** Worm burden of intestinal adults (**A**) and muscle larvae (**B**) was assessed in mice inoculated orally with *T. spiralis* ML treated by ADCC assay. The ML were incubated with 1:100 dilutions of anti-rTsSP serum and 2 × 10^5^ mouse peritoneal macrophages (PM) at 37 °C for 24 h. Three groups of mice were orally inoculated with 100 ML treated by ADCC assay. The results are shown as the mean ± SD (*n* = 6). Asterisks demonstrate a remarkable difference (*P *< 0.01) in parasite burdens of the anti-rTsSP serum group relative to those in the normal serum group.

ADCC results showed that anti-rTsSP serum promoted the adherence of PM to NBL and ML (Figures 10, 11). When 1:100 dilutions of anti-rTsSP serum was supplemented and incubated for 72 h at 37 °C, the ADCC caused a significant death of NBL and ML (52 and 44.67% cytotoxicity, respectively), in comparison with the larvae treated by pre-immune serum (21.33%, $\upchi^{ 2}_{\text{NBL}}$ = 271.493; 18%, $\upchi^{ 2}_{\text{ML}}$ = 57.520, *P* < 0.01). The cytotoxicity was the dose-dependent of anti-rTsSP antibody (*r*_NBL_ = − 0.980, *r*_ML_ = − 0.961, *P* < 0.01), which had a decreasing trend following serum dilution elevation (*F*_NBL_ = 171.618, *F*_ML_ = 60.175, *P* < 0.05). There was a significant positive correlation between cytotoxicity and culture time (*r*_NBL_ = 0.989, *r*_ML_ = 0.983, *P* < 0.01); cytotoxicity shows an increasing trend with the prolongation of culture time (Figure 12) (*F*_NBL_ = 148.587, *P* < 0.01; *F*_ML_ = 144.543, *P *< 0.01). The mice inoculated orally with ML treated by ADCC assay with anti-rTsSP serum exhibited a 89.40% reduction of intestinal AW at 3 dpi and a 36.50% reduction of ML at 42 dpi (Figure 13), when compared with ML treated by pre-immune serum (*F*_adults_ = 146.561, *P* < 0.01; *F*_larvae_ = 6.255, *P *< 0.01). These results suggest that specific anti-rTsSP antibodies could obviously kill the ML and decrease its infectivity by an ADCC fashion.

### Immune responses triggered by rTsSP

**Figure 14:**
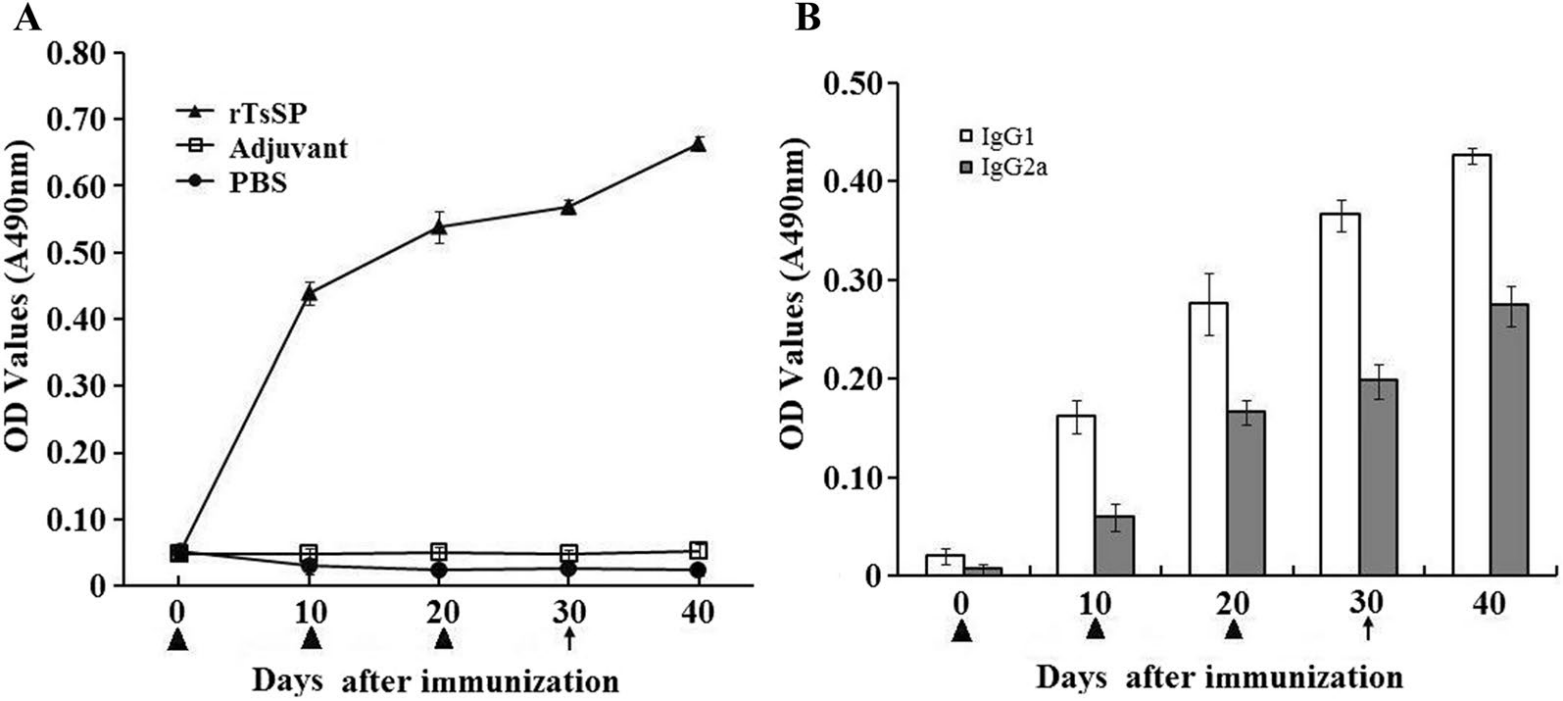
**Analysis of anti-rTsSP IgG responses in mice vaccinated with rTsSP. A** Anti-rTsSP IgG levels in sera of immunized mice at diverse time intervals after immunization. **B** The IgG subclass response of immunized mice was observed at diverse time intervals after immunization. The OD values of each group are the mean ± SD of 10 mice. The vaccination times are marked as a triangle, and challenge infection time is marked as an arrow.

In order to evaluate the humoral immune responses to rTsSP, ELISA was utilized to assay anti-rTsSP antibodies (IgG, IgG1, and IgG2a) at different intervals after immunization. The results show that anti-rTsSP IgG level of mice vaccinated with rTsSP was distinctly elevated after the second and third vaccination, but anti-rTsSP IgG was not detected in mice injected with adjuvant or PBS (Figure 14). The level of IgG1 on days 10, 20, 30 and 40 after vaccination was obviously higher than those of IgG2a (*t*_10days_ = 13.277, *t*_20days_ = 9.146, *t*_30days_ = 20.058, *t*_40days_ = 19.734, *P* < 0.05), demonstrating that the dominating IgG subclass triggered by rTsSP was IgG1, and Th2-predominant immune response was elicited, but the IgG2a antibody response was also induced after the second immunization with rTsSP.

### Immune protective effects of rTsSP

**Figure 15:**
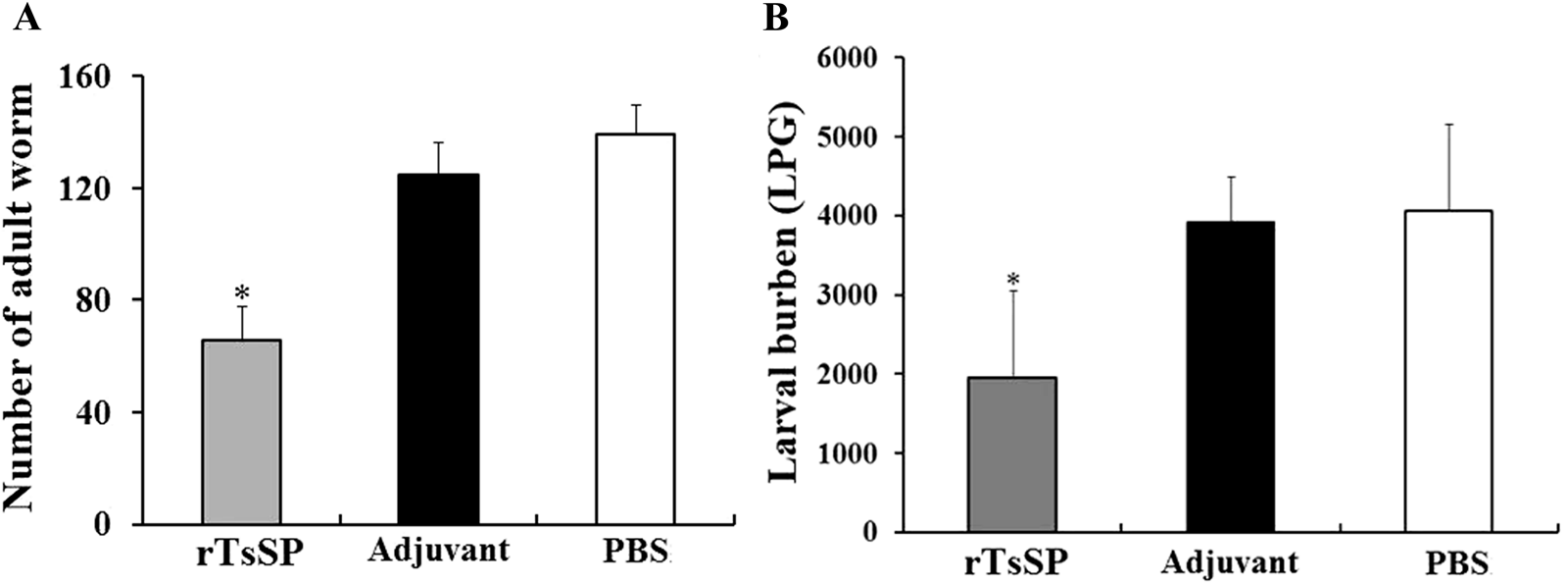
**Worm burdens of intestinal adults (A) and muscle larvae (B) recovered from three groups of mice after being infected with 300 muscle larvae of** ***T. spiralis*.** Results are expressed as the mean ± SD of 10 mice per group. Asterisks demonstrate an obvious difference (*P *< 0.01) in terms of worm burdens from rTsSP-immunized mice in comparison with the adjuvant and PBS groups.

The mice immunized with rTsSP produced a 52.70% reduction of intestinal adult worm burden at 5 dpi and a 52.10% reduction of muscle larval burden at 42 dpi (Figure 15), in comparison with the PBS control group (*F*_adult_ = 55.914, *F*_larva_ = 12.612, *P *< 0.01). Moreover, the adult and larval burden of immunized mice was also significantly lower than those of the adjuvant-injected group alone (*P *< 0.01).

## Discussion

Serine protease is a superfamily of proteolytic enzymes and has a wide variety of biological functions in parasite infection [46]. They are involved in larval invasion, molting, digestion and proteolysis [47, 48]. A previous study showed that a serine protease with “trypsin-like” activities, which was from *Schistosoma mansoni* cercariae secretory products, was related with the host’s skin invasion [49]. A neutral elastase from *Onchocerca volvulus* ES products degraded host’s dermal components (extracellular matrix, laminin, collagen type IV and fibronectin), suggesting that the elastase was related to the degradation of the host’s elastic fibres [50]. An elastolytic enzyme was found in ES products of *Ancylostoma caninum* third-stage larvae. This elastolytic enzyme could prevent fibrin clot formation and promote fibrin clot dissolution [51]. Serine proteases secreted from nematode *Trichuris muris* degraded the intestinal main mucin (Muc2), depolymerized the mucin network and destroyed the mucus barrier [52].

Some serine proteases (e.g., TspSP-1 and trypsin-like 45 kDa antigen) have been identified in *T. spiralis* ML and AW ES products [53, 54], and the enzymic activity of serine protease was detected in *T. spiralis* ML and AW ES products [55, 56]. When the IIL were co-cultured with IEC, the serine protease expression level in IIL was obviously elevated relative to ML [57]. The monoclonal antibodies against serine proteases TspSP-1 inhibited the larval invasion of IEC and migration, suggesting that TspSP-1 might exert a pivotal function for degrading intercellular or cytoplasmic proteins, consequently facilitating the larval invasion [53]. Anti-TspSP-1.2 serum partially prevented the larval invasion of IEC, and the rTspSP-1.2 protein elicited an immune protection in immunized mice [21]. Another *T. spiralis* serine protease (Ts-Adsp) was screened from an adult *T. spiralis* cDNA library, and the mice immunized with rTs-Adsp produced a 46.5% larval burden reduction relative to the control group [58]. Mice immunized with the DNA vaccine from serine protease of *T. spiralis* NBL showed a 77.93% larval reduction after challenge with the ML [13]. These results suggest that serine proteases might be connected with intestinal mucosa invasion and could be potential vaccine targets against *Trichinella* infection.

In this study, the TsSP transcription was detected by qPCR at different phases of *T. spiralis* (NBL, ML, IIL and AW) and the transcription level was not statistically different among different stages. The TsSP expression was observed by IFT at various stages of this nematode, and located in cuticles and whole worm bodies of NBL, ML, IIL, AW and embryos. Other serine proteases of *T. spiralis* were also expressed in various stages of *T. spiralis* [26, 59]. The results of Western blot analysis demonstrated that several native TsSP proteins in *T. spiralis* ES and soluble proteins could be recognized by anti-rTsSP serum, suggesting that the TsSP might have different isoforms, or the TsSP was one member of the serine protease family which has the same functional domains, or the TsSP could possibly be processed by alternative splicing or post-translational modifications [42, 60]. The results suggest that the serine proteases had housekeeping functions and might be an obligatory protein for *T. spiralis* larval growth and development.

Far Western blotting has been widely employed in numerous studies on protein–protein interactions [61]. The interaction between rTsSP and IEC was also identified in the present study. Far Western results indicate that anti-rTsSP serum identified about 24 bands of IEC proteins incubated with rTsSP, indicating the rTsSP bound to IEC. The results of IFT and confocal microscopy show that rTsSP can specifically bind to the IEC membrane and enter the cytoplasm. The binding between rTsSP and IEC was the protein dose-dependent as shown in Figure 6. The results suggest that rTsSP could bind with several IEC proteins [62]. Furthermore, anti-rTsSP antibodies could inhibit the larval invasion of enterocytes and the inhibition was antibody dose-dependent. When the 1:50 dilutions of different serum were used, the inhibition of infection serum on the larval invasion was more evident than those of anti-rTsSP serum. It is likely that the antibodies against other *T. spiralis* proteins (e.g., glutathione S-transferase, nudix hydrolase) in infection sera also took part in inhibition of larval invasion [30, 42, 48, 63]. The results indicate that there was a protein–protein interaction between TsSP and IEC, and TsSP might play an important part in larval invasion of the host’s intestinal epithelium. Nevertheless, the interaction mechanism of TsSP and IEC should be studied in future experiments.

Anti-*Trichinella* antibodies killed *T. spiralis* larvae through an ADCC mode [42, 43]. Our results from the ADCC test indicate that anti-rTsSP antibodies participated in the killing of *T. spiralis* NBL and ML. Peritoneal macrophages adhered to and damaged the larvae with the aid of anti-rTsSP serum, and the killing was also antibody dose-dependent. When the mice were inoculated orally with ML treated by ADCC, the reduction of intestinal adult worms reached up to 89.40%. The results indicate that anti-rTsSP antibodies significantly killed the ML, decreased larval infectivity, and impeded larval development in the host by an ADCC mechanism [30]. These results further revealed that TsSP could be an essential protease for invasion, development and survival of this nematode.

Th2-predominant immune responses were successfully induced by vaccination of mice with rTsSP. After being orally infected by *T. spiralis* ML, the mice immunized with rTsSP displayed a 52.70% reduction of intestinal adult worm burden at 5 dpi and a 52.10% muscle larval reduction at 42 dpi. The worm burden reduction is similar with those of previous studies [26, 32, 38, 45]. The results demonstrate that the immunization of mice with an individual recombinant *Trichinella* protein could only elicit a partial immune protection against challenge infection. Besides, the immune protection of TsSP should be investigated in a domestic pig animal model. In addition, polyvalent oral vaccines against enteral invasive stages of *T. spiralis* need to be developed in further studies [11, 64, 65].

In summary, our results reveal that TsSP is expressed at diverse *T. spiralis* phases. The rTsSP can specifically bind to and interact with host’s IEC. Anti-rTsSP antibodies inhibited the larval invasion of the host’s enterocyte in a dose-dependent manner. Anti-rTsSP antibodies also killed the *T. spiralis* NBL and ML, decreased larval infectivity and development in the host by an ADCC mode. The vaccination of mice with rTsSP produced Th2 predominant immune response and a partial immune protection against challenge infection. The results suggest that TsSP might be an indispensable protease for invasion and development of this nematode. It is likely a potential vaccine target against enteral *Trichinella* infection. But, polyvalent oral vaccines against enteral *T. spiralis* stages need to be further developed.

## Abbreviations

ADCC: antibody-dependent cellular cytotoxicity
AW: adult worms
BSA: bovine serum albumin
DAPI: 4′,6-diamidino-2-phenylindole
dpi: days post-infection
ES: excretory–secretory
FCA: Freund’s complete adjuvant
FIA: Freund’s incomplete adjuvant
G3PDH: glyceraldehyde 3-phosphate dehydrogenase
hpi: hour post-infection
ICT: International Commission on Trichinellosis
IEC: intestinal epithelial cell
IFT: immunofluorescent test
IIL: intestinal infective larvae
IPTG: isopropyl β-d-1-thiogalactopyranoside
LPG: larvae per gram
ML: muscle larvae
MPM: mouse peritoneal macrophages
NBL: newborn larvae
OPD: *o*-phenylenediamine dihydrochloride
ORF: open reading frame
PI: propidium iodide
qPCR: quantitative PCR
TBS: tris-buffered saline
TsSP: *Trichinella spiralis* putative serine protease
